# African swine fever virus infection in Classical swine fever subclinically infected wild boars

**DOI:** 10.1186/s12917-017-1150-0

**Published:** 2017-08-01

**Authors:** Oscar Cabezón, Sara Muñoz-González, Andreu Colom-Cadena, Marta Pérez-Simó, Rosa Rosell, Santiago Lavín, Ignasi Marco, Lorenzo Fraile, Paloma Martínez de la Riva, Fernando Rodríguez, Javier Domínguez, Llilianne Ganges

**Affiliations:** 1 IRTA, Centre de Recerca en Sanitat Animal (CReSA, IRTA-UAB), Campus de la Universitat Autònoma de Barcelona, 08193 Bellaterra, Spain; 2 Servei d’Ecopatologia de Fauna Salvatge, Departament de Medicina i Cirurgia Animals, Universitat Autònoma de Barcelona, 08193 Bellaterra, Spain; 3 OIE Reference Laboratory for Classical Swine Fever, IRTA-CReSA, Campus de la Universitat Autònoma de Barcelona, 08193 Barcelona, Spain; 4 Departament d’Agricultura, Ramaderia, Pesca i Alimentació (DARP), Centre de Recerca en Sanitat Animal (CReSA, IRTA-UAB), Campus de la Universitat Autònoma de Barcelona, 08193 Bellaterra, Spain; 5 Departament de Producció Animal, ETSEA, Universidad de Lleida, 25198 Lleida, Spain; 6 Departamento de Biotecnología, Instituto Nacional de Investigación y Tecnología Agraria y Alimentaria (INIA), 28040 Madrid, Spain

**Keywords:** CSFV, CSF postnatal persistent infection, Subclinical CSF, ASFV, Wild boars, Viral load, Innate immune response, Adaptive immune response, Disease

## Abstract

**Background:**

Recently moderate-virulence classical swine fever virus (CSFV) strains have been proven capable of generating postnatal persistent infection (PI), defined by the maintenance of viremia and the inability to generate CSFV-specific immune responses in animals. These animals also showed a type I interferon blockade in the absence of clinical signs. In this study, we assessed the infection generated in 7-week-old CSFV PI wild boars after infection with the African swine fever virus (ASFV). The wild boars were divided in two groups and were infected with ASFV. Group A comprised boars who were CSFV PI in a subclinical form and Group B comprised pestivirus-free wild boars. Some relevant parameters related to CSFV replication and the immune response of CSFV PI animals were studied. Additionally, serum soluble factors such as IFN-α, TNF-α, IL-6, IL-10, IFN-γ and sCD163 were analysed before and after ASFV infection to assess their role in disease progression.

**Results:**

After ASFV infection, only the CSFV PI wild boars showed progressive acute haemorrhagic disease; however, the survival rates following ASFV infection was similar in both experimental groups. Notwithstanding, the CSFV RNA load of CSFV PI animals remained unaltered over the study; likewise, the ASFV DNA load detected after infection was similar between groups. Interestingly, systemic type I FN-α and IL-10 levels in sera were almost undetectable in CSFV PI animals, yet detectable in Group B, while detectable levels of IFN-γ were found in both groups. Finally, the flow cytometry analysis showed an increase in myelomonocytic cells (CD172a^+^) and a decrease in CD4^+^ T cells in the PBMCs from CSFV PI animals after ASFV infection.

**Conclusions:**

Our results showed that the immune response plays a role in the progression of disease in CSFV subclinically infected wild boars after ASFV infection, and the immune response comprised the systemic type I interferon blockade. ASFV does not produce any interference with CSFV replication, or vice versa. ASFV infection could be a trigger factor for the disease progression in CSFV PI animals, as their survival after ASFV was similar to that of the pestivirus-free ASFV-infected group. This fact suggests a high resistance in CSFV PI animals even against a virus like ASFV; this may mean that there are relevant implications for CSF control in endemic countries. The diagnosis of ASFV and CSFV co-infection in endemic countries cannot be ruled out and need to be studied in greater depth.

## Background

African swine fever (ASF) and Classical swine fever (CSF) are highly contagious viral diseases of domestic pigs, wild boars and feral pigs [1, 2]. Because of their high socioeconomic impact, both diseases must be reported to the World Organisation for Animal Health (OIE).

ASF is caused by the large DNA virus African swine fever virus (ASFV), currently the only member of the *Asfarviridae* family [3]. ASFV encodes between 150 and 167 proteins, a number of which have been shown to modulate host immune responses [1, 4]. Since 2007, a highly virulent ASFV has spread from Sub-Saharan Africa to Eastern Europe being detected in Russia, Armenia, Azerbaijan, Estonia, Ukraine and Lithuania, among other places [5]. Direct contact between infected wild boars and domestic pigs has played a relevant role in the spread of the disease in the Caucasus region and the Russian Federation [5–8]. CSF remains endemic in areas of Asia, Europe, Central and South America and parts of Africa. The disease is caused by Classical swine fever virus (CSFV) which belongs to the Pestivirus genus within the *Flaviviridae* family [9]. CSFV is composed of a lipid envelope, a capsid and a single plus-strand RNA genome carrying a single, large open reading frame (ORF) flanked by two untranslated regions (UTRs) [10].

Both ASFV and CSFV share a tropism for immune system cells, mainly those that are derived from the monocyte-macrophage lineage, which are central in orchestrating innate and adaptive immune responses [1, 11, 12]. It is assumed that the infection of these cells plays an important role in virus replication and pathogenesis by exploiting their migratory ability, which promotes viral spread and persistence in the host for both ASFV and CSFV.

Type I IFN-α has beneficial effects for viral infections because it restricts viral dissemination, although it can promote immunopathological events when released at high levels over a longer duration [13]. CSFV induces a potent IFN-α response, which is detected in the serum of CSFV acute-infected pigs. This response has been hypothesised to be related to disease severity rather than to protective immune responses [13, 14]. Likewise, previous studies have demonstrated the increase in type I IFN-α response after infection with ASFV virulent strains ([15–17]). In addition, macrophages, the main target cells for ASFV replication, produce a range of inflammatory mediators, including chemokines that play an important role in the disease’s progression [18]. Depending on many factors, including the nature of the viral strain and the age, breed and immune system of the host, varying degrees of disease severity can be observed, ranging from acute to subclinical or chronic forms in infections caused by both viruses [1, 4, 17, 19, 20]. In addition, the circulation of CSFV strains of low and/or moderate virulence has been associated with the maintenance of the virus in the field, generating unapparent forms of CSF [21–23].

Recently, it was proven that CSFV can generate postnatal persistence by infecting both newborn piglets and wild boars with either low- and/or moderate-virulence strains, respectively [24, 25]. During the 6 weeks after postnatal infection, most of the infected animals remained clinically healthy, despite persistently high virus titres in the blood, organs and body secretions. These animals were unable to mount any detectable CSFV-specific humoral or cellular immune responses [24, 25].

The superinfection exclusion (SIE) phenomenon is defined as the ability of a primary virus infection to interfere with a secondary infection caused by the same or a closely related virus [26–28]. In this regard, CSVF PI wild boars were protected from superinfection by the virulent CSFV Margarita strain, showing efficient suppression of superinfection in animals, especially in the absence of IFN-α, which might be associated with the lack of innate immune mechanisms [28]. Thus, considering the drastic effect of ASFV on the swine immune system, in the present study, we assessed the infection generated after African swine fever virus (ASFV) inoculation in CSFV PI wild boars. For this purpose, apparently healthy, CSFV postnatal PI wild boars (7-week-old) [24] were inoculated with a highly virulent ASFV strain. Some relevant parameters related to CSFV and ASFV replication and immune response were studied. Additionally, serum soluble factors including IFN-α, TNF-α, IL-6, IL-10, IFN-γ and sCD163 were analysed before and after ASFV infection to assess their role in the progression of disease in the CSFV PI animals. Additionally, virological and immunological parameters after ASFV infection in naive wild boars were also evaluated.

## Methods

### Cells and viruses

PK-15 cells (ATCC CCL 33) were cultured in Dulbecco’s Modified Eagle Medium (DMEM), supplemented with 10% foetal bovine serum (FBS), pestivirus-free, at 37 °C in 5% CO_2_. The cells were infected with CSFV 0.1 TCID_50_/cell in 2% FBS, and the virus was harvested 48 h later. Peroxidase-linked assay (PLA) [29] was used for CSFV titration following the statistical methods described by Reed and Muench [30].

The CSFV Catalonia 01 (Cat01) strain used in this study was isolated from the Spanish CSF epizootic in 2000–2001 [31]. This isolate belongs to the CSFV 2.3 genogroup [22]. The course of infection from this strain was found to be mild [31, 32]. Finally, the moderately virulent E75 ASFV strain was used [33]. This strain was isolated in 1975 in Spain and propagated in pig leucocytes [17].

### Experimental design

Two groups of three wild boars each (A and B), 7 weeks old with 6 kg average weight were used [28]. These animals were acquired from Gestión Cinegética Integral SL farm (Segovia, Spain).

Group A, wild boars 1 to 3, were postnatally CSFV PI animals; they were intranasally infected in the first 24 h after birth with 2.5 ml of 10^4^TCID_50_/ml of the CSFV Cat01 strain [24]. These wild boars remained apparently healthy at 7 weeks old, but were viraemic and lacked CSFV-specific cellular and humoral responses [24], similar to CSFV PI piglets that were infected postnatally [25].

The second group (Group B, animals 4 to 6), housed in an independent isolation unit at the BSL-3 facility of CReSA, (animals 4 to 6), was used as control; these animals tested pestivirus-free throughout the experiment.

All the wild boars were fed with a conventional piglet starter diet and pellets until the end of the trial (Startrite 100, Kwikstart and Prestarter; SCA Iberica S.A., Zaragoza, Spain) and were handled according to previous studies conducted in CReSA [24, 34]. After a 5-day acclimation period, all the animals were experimentally infected by intramuscular (i.m.) injection in the neck with a dose of 10^4^ TCID_50_ ASFV E75 strain. In previous studies, this viral dose caused acute ASF and often induced death within a week post-infection [17]. Sera and nasal and rectal swabs were collected at 0, 3, 5, 7 and 10 days post infection (dpi). Blood samples for the isolation of PBMCs were collected in EDTA tubes at 0 dpi and at the time of euthanasia (10 dpi).

A trained veterinarian recorded the clinical signs daily in a blinded manner [35]. The clinical signs compatible with CSFV and after ASFV infection were previously described ([17, 31, 36–38]). Moreover, the clinical statuses registered in both experimental groups were scored from 0 to 6 as follows: 0: no signs; 1: mild pyrexia; 2: pyrexia plus mild clinical signs; 3: mild-to-moderate clinical signs; 4: moderate clinical signs; 5: moderate-to-severe clinical signs and 6: death. For ethical reasons, the animals were euthanized when the clinical score reached 5 or developed any of the following symptoms: fall of the hindquarters, inability to drink or feed, prostration or moderate nervous disorders. After euthanasia, an exhaustive necropsy was conducted, in which the presence of pathological findings in different organs was evaluated and tissues (spleen, lung and tonsil) were obtained. Euthanasia was performed according to European Directive 2010/63/EU, using a pentobarbital overdose of 60–100 mg/kg administered via the anterior vena cava. Animal care and procedures were in accordance with the guidelines of the Good Experimental Practices (GEP), under the supervision of the Ethical and Animal Welfare Committee of the Autonomous University of Barcelona (UAB), and they were approved under number 8804, according to existing national and European regulations. Additionally, the biosafety level of the viruses used in this study was stated as biosecurity level 3, as approved by the Biosafety Committee of the UAB, with registration assignment AR-296-15.

### Detection of CSFV RNA

CSFV RNA was extracted from sera, nasal and rectal swabs, and tonsil, spleen and lung tissues using the NucleoSpin RNA isolation kit (Macherey-Nagel), according to the manufacturer’s instructions. The presence of CSFV RNA was analysed by a CSFV qRT-PCR [39]. Positive results were considered for threshold cycle values (Ct) equal to or less than 42, when fluorescence is no longer detected. Samples in which fluorescence was undetectable were considered negative.

### Detection of ASFV DNA

ASFV DNA was extracted from sera, nasal and rectal swabs and tissue samples (tonsil, spleen and lung) using the NucleoSpin blood kit (Macherey-Nagel, Düren, Germany) according to the manufacturer’s recommendations. The presence of viral DNA was analysed by ASFV qRT-PCR technique using a Universal Probe Library [40]. Positive results were considered for threshold cycle values (Ct) equal to or less than 40, when fluorescence is no longer detected. Samples in which fluorescence was undetectable were considered negative.

### Specific CSFV and ASFV antibodies detection

For CSFV-specific antibody detection, sera from 0 dpi, 7 dpi and 10 dpi were tested with neutralisation peroxidase-linked assay (NPLA) [41], and the titres were expressed as the reciprocal dilution of serum that neutralised 100 TCID_50_ of the Cat01 strain in 50% of the culture replicates. The detection of CSFV E2-specific antibodies was performed using a commercial ELISA kit (HerdChek CSFV Ab, IDEXX) following the manufacturer’s recommendations. The samples were considered positive when the blocking percentage was ≥40%. For ASFV-specific antibody detection, a blocking ELISA assay technique was conducted using a commercial ELISA kit (INGEZIM PPA Compac, Ingenasa) following the manufacturer’s recommendations. This ELISA test is validated by the European Union Reference Laboratory for ASF (EURL-ASF, CISA-INIA, Spain).

### ELISA detection of serum soluble factors

Sera collected at days 0, 7 and 10 dpi were analysed for the detection of several serum soluble factors. A house-ELISA was used to evaluate the serum IFN-α levels. Anti-IFN-α monoclonal antibodies (K9 and K17) and IFN-α recombinant protein (PBL Biomedical Laboratories, Piscataway, New Jersey, USA) were employed in the ELISA assay using a previously described protocol [14, 25, 42]. On the other hand, commercial ELISA tests were used for the detection of tumour necrosis factor-α (TNF-α) (TNF alpha ELISA Kit, Porcine, Life Technology), interleukin-10 (IL-10) (IL-10 ELISA Kit, Porcine, Life Technology), IFN-γ (IFN-γ ELISA Kit, Porcine, Life Technology) and interleuking-6 (IL-6) (IL-6 ELISA Kit, Porcine, Life Technology). The results were expressed as units per millilitre (U/ml) for IFN-α. For IFN-γ, IL-6, TNF- α and IL-10, the results were expressed in pg/ml. Finally, soluble CD-163 (sCD163) was quantified by a formerly described ELISA using lysates from CD-163-transfected Chinese hamster ovary (CHO) cells as standard [43].

### PBMC collection and phenotype analysis

To address the effect of ASFV infection on the PBMC populations in both experimental groups, a comparative study of the expression of different porcine surface markers was carried out by flow cytometry, using cells collected before and after ASFV infection (end-point lifetime after ASFV infection). PBMCs were separated from whole blood by density-gradient centrifugation with Histopaque 1077 (Sigma). The number and viability of the PBMCs were determined by staining with Trypan Blue [36]. To phenotype these cells, flow cytometry was performed using the corresponding monoclonal antibody (mAbs). The mAbs for porcine CD4 (74–12-4, IgG2b) Alexa Fluor 647 conjugate (BD Pharmingen) and CD8-α (76–2-11, IgG2a) FITC-labelled (BD Pharmingen) were used. Further, anti-CD172a mAb (BA1C11, IgG1) produced in our laboratory was used in indirect labelling, with goat anti-mouse IgG Alexa Fluor 647 conjugated (Jackson ImmunoResearch) as a secondary antibody. The staining protocols were performed as previously described [17, 25, 42]. Finally, the cells were analysed by flow cytometry, using a FACSCalibur (Becton Dickinson FACSAria I) (Becton Dickinson, San Jose, California, USA), and the positive percentages were analysed by FACSDiva software, version 6.1.2 [25, 42].

### Statistical analysis

All statistical analyses were performed using SPSS software, version 15.0 (SPSS Inc., Chicago, Illinois, USA). The significance level (α) was set at *P* < 0.05. Throughout the trial, a non-parametric test (Mann-Whitney) was chosen to compare values obtained from the clinical and virological parameters, PBMC phenotype and soluble factors parameters between Groups A and B. This non-parametric analysis was chosen due to the non-normality pattern observed for the studied parameters and the small number of animals used in each experimental group. Finally, a survival analysis was carried out to test differences in survival throughout the trial between Groups A and B.

## Results

### Clinical signs developed after E75 ASFV infection

**Fig. 1:**
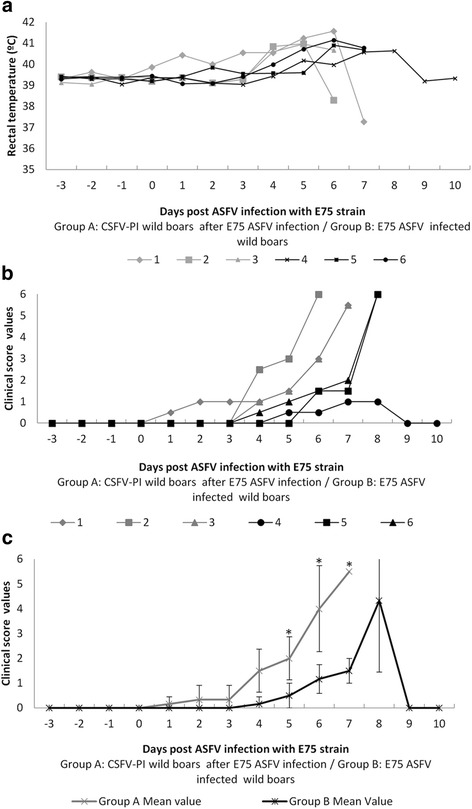
Rectal temperature and clinical score values after ASFV infection. a The individual rectal temperature values that were detected after the ASFV strain infection. Temperatures greater than 40 °C were considered fevers. b The individual clinical score values that were detected after the E75 strain infection. c The mean and standard deviation of the clinical score values recorded after ASFV infection. An asterisk indicates statistical significance between the two groups from day 5 until day 7 (*p* < 0.05)

After the E75-ASFV infection, one of the CSFV PI wild boars (CSFV-ASFV infected) from Group A showed mild clinical signs from 1 dpi to 3 dpi, consisting of mild pyrexia (40 °C - 40.5 °C) and mild apathy. No clinical signs were recorded in the pestivirus-free-ASFV infected wild boars (Group B) within this period; therefore, from 1 dpi to 3 dpi no statistical significant differences were found between both experimental groups (Fig. 1a–c). At 4 dpi, the three CSFV-ASFV infected animals from Group A presented mild apathy. Animal number 2 showed evidence of cyanosis and petechial haemorrhages in the ears and abdomen at five dpi; it suddenly died at 6 dpi. The rest of the infected wild boars from Group A showed signs of severe apathy, semi-prostration, cyanosis and haemorrhages in the skin, dyspnoea and tremors, for which reason they were euthanized at 7 dpi, reaching clinical score values from 1.5 to 5.5 (Fig. 1a–c). On the other hand, only pyrexia and mild apathy were registered from day 4 to 7 dpi in pestivirus-free-ASFV infected wild boars (Group B); nevertheless, two animals suddenly died at 8 dpi. On the contrary, only animal number 4 presented pyrexia (40 °C - 40.7 °C) and slight nasal secretions between 5 dpi and 8 dpi, remaining clinically asymptomatic from then on until the end of the experiment (Fig. 1a–c). Thus, statistical significant differences (*p* < 0.05) in terms of clinical signs between both experimental groups were found from 4 dpi to 7 dpi (Fig. 1c).

Despite the clinical differences observed between both groups, no statistical differences were found in the survival analysis. In this respect, the survival of CSFV-ASFV infected animals (Group A) was equal to the pestivirus-free-wild boars from Group B. Only animal 4 in Group B survived the ASFV infection until the end of the trial (10 dpi).

Although animals 5 and 6 of Group B suddenly died, several macroscopic lesions were observed after necropsy, which consisted of splenomegaly, splenic haemorrhagic, generalized lymphadenitis, moderate haemorrhagic enteritis, petechiae in the kidney and ascites. The surviving animal at the end of the study (animal 4 of Group B) had only a few petechiae in the kidney.

### CSFV RNA detection after ASFV infection

**Fig. 2:**
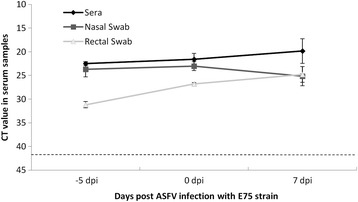
Mean values and standard deviation values of the CSFV Catalonia strain RNA detection in serum, nasal and rectal swabs at different times before and after ASFV infection. CSFV Catalonia strain RNA detection in serum, nasal and rectal swabs samples in CSFV PI animals from Group A (numbers 1 to 3) at 5 days before ASFV infection, and at 0 and 7 days post ASFV infection. A dotted bar indicates the detection limit of the technique above 42 Ct

Wild boars from Group A maintained similar CSFV RNA load during the whole trial even after ASFV infection. A high and constant CSFV RNA load in serum and swabs was found when examined by CSFV q-RT-PCR [39] (Fig. 2). A similar CSFV RNA load was detected in the tonsil and lung from these infected animals, with Ct values about 22–25 (data not shown). The samples analysed from Group B were CSFV RNA negative (data not shown).

### ASFV DNA detection

**Fig. 3:**
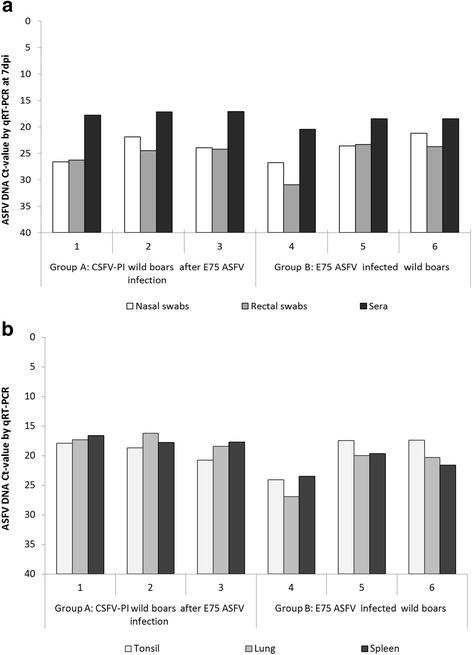
ASFV E75 strain DNA detection in serum, nasal and rectal swabs and in tissues samples at different times after ASFV infection. **a** ASFV DNA detection in serum, nasal and rectal swabs samples in CSFV PI animals from Group A (numbers 1 to 3) and in pestivirus-free animals from Group B (numbers 4 to 6) at 7 days post ASFV infection. **b** ASFV DNA detection in tonsil, lung and spleen samples in both experimental Groups A and B. The detection limit of the technique was above 40 Ct

ASFV DNA was detected in all samples analysed from both experimental groups. At 7 dpi, the animals from Group A (CSFV-ASFV infected) were found viraemic with a Ct value of around 17, corresponding with a high viral load in sera (Fig. 3a). In the case of ASFV-infected animals from Group B, similar Ct values were detected in two serum samples (animals 5 and 6). On the contrary, a lower DNA viral load (Ct value 24) was detected in the serum of animal 4. The ASFV DNA viral load detected in nasal and rectal swabs was comparable between both groups except for animal 4 (Group B), with a lower Ct value. At 10 dpi, the ASFV DNA viral load detected in the survivor (animal 4) considerably diminished with respect to day 7, with a Ct value of 26.25, 32.65 and 34.54 in sera, nasal and rectal swabs, respectively. Finally, similar results to those previously found in serum and swabs were obtained in tonsil, lung and spleen tissues in both experimental groups (Fig. 3b).

### Specific CSFV and ASFV antibodies detection

Sera from all the animals in Groups A and B were negative for the CSFV E2-specific antibodies and CSFV neutralising antibodies during the trial. For ASFV-specific antibody response, only the surviving animal of Group B (animal 4) was positive at 10 dpi, with a blocking percentage of 72%. The rest of the animals from Group B (animals 5 and 6) and from Group A remained negative at all sampling times.

### ELISA detection of serum soluble factors

**Fig. 4:**
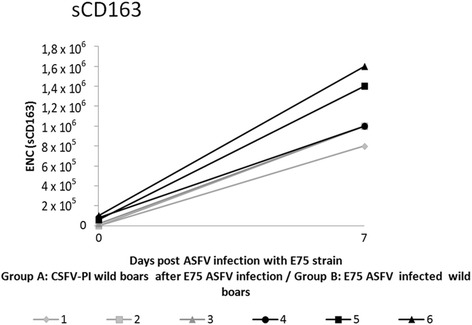
sCD163 levels found before and after 7 days post ASFV infection in both experimental Groups A and B. The results are expressed as the equivalent number of CD-163-transfected cells

TNF-α and IL-6 were not detected in any sample at 0 and at 7 days post-ASFV infection. In contrast, high IFN-α levels were found only in the samples from Group B after 7 days post-ASFV infection (Table 1), and significant differences (*p* < 0.05) were observed between Group A and B piglets at day 7 dpi. Within the B group, the IFN-α values observed for pigs 5 and 6 were approximately 30% higher than that detected in pig 4 (Table 1). For IFN-γ, a variable response was detected in both experimental groups after ASFV infection (7 dpi), as two out of three animals were positive in Group A and one out of three was positive in Group B (Table 1). Additionally, IL-10 was detected only in Group B at 7 dpi. Finally, high levels of sCD163 were detected at 7 dpi in both Groups A and B (Fig. 4). For animal 4 (Group B) the level of sCD163 decreased to basal levels at 10 ASFV dpi (under 80,000).

**Table 1:** Serum soluble factors detection at 7 days post ASFV infection

Experimental groups	Animal identifications	ELISA detection of serum soluble factors		
		IFN-α	IFN-γ	IL-10
CSFV-PI infected with ASFV E75	1	24	0	0
	2^a^	0	66	0
	3	40	915	0
Pestivirus Free- Infected with ASFV E75	4	122	0	39
	5	193	0	17
	6	184	213	149

^a^pig number 2 was sampled at 6 ASFV dpi (before necropsy)

### PBMC phenotype analysis after CSFV and ASFV infection

Before ASFV infection (0 dpi), the percentage of the T CD4^+^ cell population was higher (*p* = 0.08) in the CSFV PI animals (Group A, animals 2 and 3) than in the non-infected animals (pestivirus-free wild boars 4 to 6 from Group B). Further, the percentage of CD172a^+^ was higher in the infected than in the non-infected animals (Group B). Unfortunately, animals 5 and 6 (Group B) died suddenly, and therefore, it was impossible to collect PBMCs after ASFV infection. Thus, the comparative study of the porcine surface markers expression included samples only from animals numbered 2 to 4 at 7 dpi. The results showed a marked effect on the CD4^+^ and CD172a^+^ populations in animals from Group A (1 and 2) after ASFV infection (Table 2). On the one hand, the percentage of T CD4^+^ cell populations drastically decreased from 15 to 17% to 3% (*p* = 0.10). On the other hand, at the time of necropsy, the CSFV PI animals (Group A) showed a 20% increase in the CD172a^+^ cell population after ASFV infection compared to their pre-infection status. Moreover, the percentage of CD172a^+^ cells in these animals 2 and 3 at 7 dpi was higher and statistically different (*p* = 0.08) than in the non-infected naive animals (wild boars from group B before ASFV infection). In contrast, the surviving ASFV-infected animal from Group B at 10 dpi (animal 4) presented around a 10% decrease in the CD172a^+^ compared to the samples at day 0 (Table 2). In parallel, the forward versus scattered dot-plot side representation (FSC/SSC) indicated a high-complexity region that may correspond to immature granulocytes in the samples from Group A (data not shown).

**Table 2:** PBMC phenotype analysis after CSFV and ASFV infection

Experimental groups	Animal identification	Timing post-ASFV-infection					
		0 days post-ASFV-infection^a^			End-point lifetime after ASFV infection^a^		
		CD4^+^	CD8^+^	CD172a^+^	CD4^+^	CD8^+^	CD172a^+^
CSFV-PI infected with ASFV E75	2	17	17	43	3	11	76
	3	15	14	63	3	21	73
Pestivirus Free- Infected with ASFV E75	4	7	16	30	11	26	19
	5	8	17	27			
	6	7	20	32			

^a^Values expressed in %

## Discussion

The ASF and CSF situation in Eastern Europe is of concern due to the current epidemiological complexity [7, 24, 25]. In this sense, the role of wild boar populations may be one of the main components in disease maintenance in these geographic areas [7, 24].

In the present work, we studied some virological and immunological parameters related to the innate and adaptive immune response in CSFV PI animals after ASFV infection, a virus that causes one of the most devastating diseases in swine. Likewise, we assessed the survival and disease progression in animals with CSF PI compared to those with ASFV infection. For this purpose, CSFV-PI wild boars were inoculated with a virulent ASFV strain. Finally, virological and immunological parameters after ASFV infection in naive wild boars were also assessed.

Even though the high infection rate of the Cat01 strain of CSFV PI wild boars was in accordance with previous studies, good health status was maintained before ASFV infection [24, 28]. Interestingly, the animals infected with a CSF persistent form (Group A) showed a progressive acute haemorrhagic disease after ASFV infection. In Group B, two pestivirus-free wild boars suddenly died shortly after ASFV infection (5 and 6 dpi), accordingly with previously published reports [44]. However, none of the pestivirus-free-ASFV infected wild boars (Group B) developed the haemorrhagic clinical form of the disease. Despite the different clinical pictures recorded between both experimental groups, the survival rate after ASFV infection was similar, suggesting that CSFV PI animals, regardless of their immunosuppression state, are equally susceptible towards ASFV infection as pestivirus-free animals would be [24, 25].

Notably, the ASFV DNA load detected after infection in sera, swabs and tissue samples was high and similar for both groups, correlating with the ASF acute form [17, 44]. In addition, the CSFV RNA load in CSFV-ASFV-infected animals (Group A) remained unaltered after ASFV infection during the trial [24, 42].

A recent report showed that vaccination against CSFV could yield CSF progression in CSFV PI animals, although signs of cyanosis or haemorrhage were not previously observed [42]. Considering this precedent, the ASFV infection could be a trigger factor for the haemorrhagic disease progression in the CSFV PI wild boars. It is worth highlighting the high variability in the infection generated by ASFV in swine [37]. Although only three animals per group were used in this study, our results suggest that the CSFV subclinical infection may also predispose animals to ASF disease and aggravate its progression, considering that the three animals from Group A showed cyanosis and severe haemorrhage in the skin. In agreement with the high variability generated by ASFV in swine, two of the pestivirus-free-ASFV infected wild boars from Group B suddenly died and one of them remained clinically asymptomatic until the end of the experiment. Although clinical signs were almost unapparent, this animal remained ASFV positive in the serum and excreted virus during the study. In addition, it was the only animal able to seroconvert against ASFV at 10 dpi. It is known that pigs that survive natural infection usually develop antibodies against ASFV from 7 to 10 days post-infection which persist for a long period of time [45]. Therefore, the detection of specific antibodies against ASFV is very useful for the diagnosis of inapparent forms of ASF [46]. Thus, despite the short duration of the experiment, this animal would represent an ASF subclinical case.

It is known that ASFV activates monocytes/macrophages that secrete a wide range of mediators including pro-inflammatory cytokines such as IL-6 and TNF-α [47–49], which can trigger acute phase reactions, inflammation, activation of endothelial cells and apoptosis. Paradoxically, the absence of IL-6 and TNF- α was detected in the sera from both experimentally infected groups. On the other hand, it is also known that IL-10 inhibits a broad spectrum of immune responses, including the suppression of T cell proliferation, cytokine production and B cell responses [50–53]. Interestingly, all the ASFV-infected wild boars from group B showed detectable IL-10 levels in sera, a finding previously described in the ASF disease [48, 54]. In contrast, despite the ASFV replication in CSF-persistent animals, IL-10 levels in sera were not found, as previously reported in CSF-persistent disease [25].

Notably, the sCD163 is an activation macrophage marker that has been associated with acute ASF disease at 7 dpi [17]. Accordingly, high levels were found associated with ASFV infection in both experimental groups, with the highest values detected in the Group B animals that suddenly died. Interestingly, low levels of sCD163 were found in the survivor animal from Group B at 10 dpi, at which time a lower DNA ASFV load was detected.

In parallel, we analysed the capacity of ASFV to generate IFN-γ response in both infected groups. Notably, soluble IFN-γ was detected in some animals from both experimental groups. This finding is supported by data previously published in ASF-acute-infected domestic pigs [55] and suggests the swine immune system capability in CSFV PI animals to produce Type II IFN-γ against the unrelated viral infection. Otherwise, in the case of innate immune response against ASFV, as measured by type I IFN-α in the serum, it is worth mentioning that IFN-α was detected in all ASFV-infected animals from Group B, mainly in the animals that suddenly died. In this context, the IFN-α increase has been related to the cytokine storm after ASF disease [16, 17, 56]. Remarkably, lower IFN-α levels were found in the surviving ASFV-infected wild boar, correlating with its capability to control the infection. Strikingly, once again, the IFN-α response seemed to be impaired in CSFV PI animals, even after infection with the ASFV virus, which induces a potent effect on the innate immune system. The CSFV PI animals remained IFN-α negative, a cornerstone in the innate immune mechanisms; this fact might promote the maintenance of a high and constant CSFV load, as already described [28, 42]. Additionally, it may explain the CSFV ability to generate the SIE phenomenon previously described in swine [28].

The flow cytometry analysis showed that the percentage of CD8+ T cell populations were similar between both experimental groups before ASFV infection. Whereas, CD4^+^ T cell populations were increased in CSFV PI animals, despite previously being in a state of immunosuppression [24, 25]. However, the CD4^+^ T cell subset drastically decreased after ASFV infection, suggesting the incapacity to induce an effective immune response to ASFV [44, 57]. These results are underpinned by the capacity to overcome the disease in the ASFV survivor animal from Group B, in which the T cell populations remained almost within normal parameters. Finally, it is known that myeloid cells, granulocytes and monocyte/macrophages play a relevant role in ASFV and CSFV pathogenesis in swine. Considering this fact and in accordance with a previous report, the percentage of CD172a^+^ cells found in the PBMCs from CSFV PI animals increased by 20% over the value found in naive wild boars [25]. It is also known that the progression of CSFV infection induced a clear imbalance in the proportion of blood cells towards enrichment in the CD172a^+^ cells subset [36, 58]. This population, which has been identified as immature granulocytes [36], specifically 6D10^+^ cells, were the predominant cell population in CSFV PI pigs [25]. Thus, in agreement with previous data, the 6D10^+^ cell profile can explain the increase in the CD172a^+^ cells after ASFV infection, due to the constant imbalance in the homeostasis in CSFV-ASFV infected wild boars, thereby hampering the immune response against ASFV. On the contrary, the lowering of CD172a^+^ cells in ASFV-infected animal 4 correlates with the decrease in macrophages described after ASFV infections [57, 59].

## Conclusion

Overall, our findings showed the development of a cellular homeostasis imbalance, and a type I Interferon blockade after ASFV infection of CSFV PI animals, which may explain how CSFV persists in the infected host. On the other hand, it should be noted that in terms of viral replication, ASFV infection does not produce any interference with CSFV replication, or vice versa. Despite the high DNA load generated after the ASFV infection, in CSFV PI animals, the CSFV RNA load remained high and unaltered during the trial. Additionally, ASFV infection could be a trigger factor for disease progression in the CSFV PI wild boars. Likewise, our results also suggest that the subclinical CSFV infection may be aggravating the ASF disease, considering that only the three CSFV persistently infected animals developed clinical haemorrhagic signs after ASFV infection. The survival rate against ASFV in CSFV PI animals was equal to those animals in the pestivirus-free ASFV-infected group; this fact may mean that there are relevant implications for CSF control in endemic countries, because they suggest the high resistance of these types of animals, even against a virus like ASFV. Finally, considering the co-existence of the increasing spread of ASF and the presence of CSF subclinical forms in endemic countries for both diseases, the possibility of ASFV and CSFV co-infection in swine cannot be ruled out and needs to be studied in greater depth.

## Abbreviations

ASFV: African swine fever virus
BSL3: Biosecurity level 3
CD: Cluster of differentiation
CHO cells: Chinese hamster ovary cells
CSFV: Classical swine fever virus
DMEM: Dulbecco’s Modified Eagle Medium
DNA: Deoxyribonucleic acid
FBS: Foetal bovine serum
FITC: Fluorescein isothiocyanate
IFN: Interferon
Ig: Immunoglobulin
IL: Interleukin
mAb: Monoclonal antibody
PI: Persistently infected
PLA: Peroxidase-linked assay
qRT-PCR: Quantitative reverse transcription polymerase chain reaction
RNA: Ribonucleic acid
